# Multiscale 3D Curvature Analysis of Processed Surface Textures of Aluminum Alloy 6061 T6

**DOI:** 10.3390/ma12020257

**Published:** 2019-01-14

**Authors:** Tomasz Bartkowiak, Christopher A. Brown

**Affiliations:** 1 Institute of Mechanical Technology, Poznan University of Technology, ul. Piotrowo 3, 60-965 Poznan, Poland; 2 Surface Metrology Laboratory, Worcester Polytechnic Institute, Worcester, MA 01609, USA; brown@wpi.edu

**Keywords:** surface, texture, machining, multiscale, aluminum alloy 6061 T6

## Abstract

The objectives of this paper are to demonstrate the viability, and to validate, in part, a multiscale method for calculating curvature tensors on measured surface topographies with two different methods of specifying the scale. The curvature tensors are calculated as functions of scale, i.e., size, and position from a regular, orthogonal array of measured heights. Multiscale characterization of curvature is important because, like slope and area, it changes with the scale of observation, or calculation, on irregular surfaces. Curvatures can be indicative of the topographically dependent behavior of a surface and, in turn, curvatures are influenced by the processing and use of the surface. Curvatures of surface topographies have not been well- characterized yet. Curvature has been used for calculations in contact mechanics and for the evaluation of cutting edges. Manufactured surfaces are studied for further validation of the calculation method because they provide certain expectations for curvatures, which depend on scale and the degree of curvature. To study a range of curvatures on manufactured surfaces, square edges are machined and honed, then rounded progressively by mass finishing; additionally, a set of surfaces was made by turning with different feeds. Topographic measurements are made with a scanning laser confocal microscope. The calculations use vectors, normal to the measured surface, which are calculated first, then the eigenvalue problem is solved for the curvature tensor. Plots of principal curvatures as a function of position and scale are presented. Statistical analyses show expected interactions between curvature and these manufacturing processes.

## 1. Introduction

The objectives of this paper are to demonstrate the viability, and to validate, in part, how surface topographies can be characterized by curvature tensors calculated from areal topographic measurements of manufactured surfaces. In addition, two methods for specifying the scale are studied. The machined, honed, and mass finished surfaces have regular and irregular topographic components. The second-order curvature tensors vary with scale, position, and orientation, i.e., direction. They are calculated from regular arrays of measured surface heights, producing multiscale characterizations that are both position- and orientation-specific. The validation is tested by comparing the results with expectations, based on the machining, honing, and finishing processes.

Appropriate characterization of topographies is essential for discriminating with confidence surfaces with topographies that were created differently or that behave differently, and for discovering strong correlations between processing and topographies, or between topographies and behavior. The value of surface metrology for product and process design, i.e., the measurement and analysis of surface topographies, is largely founded on these abilities to discriminate and correlate [1,2].

Topographies can have components that are regular, like form, and components that are irregular, i.e., roughness. Sometimes the term roughness is used simply to refer to fine-scale topographies, even if they are highly regular, e.g., certain engineered surfaces. Surfaces that are essentially regular might be sufficiently characterized by a few measurements and parameters. Irregular surfaces can require millions of height measurements, multiscale geometric analyses, and statistics for sufficient characterizations [2].

Curvature is particularly attractive as a characterization method. Curvature is approximately the spatial derivative of the slope. No datum is required for the characterization of curvature, unlike heights or slopes. This aspect of curvature characterization can be especially valuable when the datum is not obvious, such as in characterizing redundant surfaces or voids, which can be measured with tomography.

Curvature is an essential parameter for characterizing edges and surfaces. The curvature of cutting edges has been discussed in the literature [3,4,5]. Curvature of peaks as a geometric property of surfaces is important in contact mechanics (e.g., [6,7,8]). Characterizing the valleys of topographies by their curvature also could be important for understanding crack initiation, fluid retention, and adhesion. Vulliez et al. presented a strong functional correlation (R² = 0.96) at a specific scale (610 μm) between the curvature of machined surfaces and their fatigue limit [9]. Logically, some kind of multiscale characterizations of curvatures of topographies could be used for discrimination and for correlation with processing and behavior, as has been done with area-scale analysis [10]. This characterization could be useful for surface research and for product and process design. 

It is important to understand how the many ways of implementing multiscale in the analyses of characterization parameters can influence the results. Two methods are used here, and the results are compared.

Curvature has previously been calculated from profile measurements as a function of scale and position, where the height, *z*, as a function of position, *x*, such that *z* = *z*(*x*) [9]. One method uses Heron’s formula to calculate the curvature, based on three points from the profile. The scale is represented by the spacing in *x* of the three height measurements selected for the calculation. The curvature can vary with position and with scale along the profile.

Here it is shown how curvature can be calculated from areal measurements, i.e., on regular orthogonal arrays of heights, *z*, in *x* and *y* such that *z = z*(*x*,*y*). Curvature can be characterized as a second-order tensor that can vary with position and scale. This is more complicated than calculating curvature from a profile, and the results can be more valuable. The result of an eigenvalue calculation gives the values and orientations of the maximum and minimum curvatures as a function of position and scale. In addition to the curvatures themselves, these results can be used to characterize the anisotropy, or directionality, of a surface, based on curvature orientation [11].

A study of the commonly used techniques for curvature estimation was presented by Petitjean [12]. Recent studies tend to concentrate on the triangular meshes because they are commonly used in representations in many computer-assisted design (CAD) or graphics programs. Many [13,14,15] use piecewise surface approximations, e.g., Bezier, quadratic, or polynomial. Thiesel et al. introduced a robust method for calculating the curvature tensor, based on vectors normal to the surface, which does not involve local surface interpolation [16]. That method calculates a curvature tensor with components that are constant within a certain triangular region on the surface. Coeurjolly et al. described a novel class of estimators of digital shapes, which are based on integral invariants [17]. They used a local approximation to convert discrete height data into continuous functions. An interesting study was presented recently by Foorginejad and Khalili, in which they introduced a method named umbrella curvature [18], which involved normal vectors and vectors between the point of estimation and their neighbors in order to estimate local curvature. Lai et al. described a method that connects profile and surface curvature. They searched principal curvatures by calculating profile curvature in multiple directions and looking for maximum and minimum values [19]. 

The physical determination of the height at a point on a real surface is problematic. During surface measurements, discrete heights are determined over lateral, or spatial, sampling zones, rather than at points. The height at a mathematical point, which is infinitesimally small, cannot be measured on a surface. Measured heights are determined at a certain lateral spacing, or sampling intervals, which might or might not exceed the size of the sampling zone. Nonetheless, the measured heights over zones are treated here, for the calculation, as an array of mathematical points, *z* = *z*(*x*,*y*). 

The actual, measured, areal surface, although continuous by the general definition of a surface, could represent an actual surface that is nowhere differentiable. However, digital representations of measured surfaces are commonly approximated as smooth. This approximation facilitates characterization by a series of curvatures that can vary with position and scale. 

In the following development of the curvature calculation, a surface will be considered to consist of a collection of heights at points on a regular, spatial grid. The surface will be considered differentiable at the scales and locations required for the calculations of normal vectors to patches, or defined regions, on the surface.

The approach here is to use a representation of areal surfaces based on heights used to calculate vectors normal to the surface. The curvature tensor is considered constant over the size of the region that represents the scale. This method for curvature estimation uses normal vectors at points that represent the center of patches, over which the normal vectors are calculated. 

Three ways to compute the normal vectors are given in [20]:

1. Covariance matrix, which computes the unit normal vectors from the neighboring points about a central point.
2. Average areal gradient, which uses horizontal and vertical differences of neighboring points around the central point.
3. Average depth change, which calculates horizontal and vertical differences from averaged neighbors.

In this work, the first method, covariance matrix, is used, because of its small estimation error in multiscale applications and the simplicity of implementation [21].

The characteristics of aluminum alloy 6061-T6, used in this study, may lead to the formation of built-up edge (BUE) when cut. This phenomenon increases the mechanical load on the cutting edge, making efficient chip flow difficult and the chip-removal process inefficient. Alloying elements (in particular, silicon present in this aluminum alloy), and the treatment methods, influence the machining properties [22,23]. The influence of machining parameters on the resulting surface texture for hard-to-cut materials was analyzed by Krolczyk et al. [24] and Twardowski et al. [25].

## 2. Materials and Methods 

### 2.1. Preparation and Measurement of the Surfaces

Two different sets of surfaces were manufactured from aluminum alloy (6061 T6), the surface topographies were measured, and the measurements were analyzed. The topographies were measured with an Olympus LEXT 4100 OLS laser scanning confocal microscope (Olympus Corporation Shinjuku, Tokyo, Japan) with a 50× objective (NA 0.93). The measurement regions were cropped to 0.11 mm × 0.11 mm for the turned and 0.075 mm × 0.075 mm for the edge. The sampling interval is about 250 nm.

One set of surfaces was manufactured by turning a rod, initially 25.45 mm in diameter, on a Haas SL10 CNC lathe (Haas Automation, Inc., Oxnard, CA, USA), first to a diameter of 24.29 mm, then to 22.30 mm in a final pass, making final cutting depth, *a_p_*, equal to 0.995 mm. The feeds were 0.2, 0.1, 0.05, 0.01 mm/rev. The spindle speed was 1000 rpm. Kennametal carbide inserts (VNMG 160404ms, KC5525, Kennametal Inc., Pittsburgh, PA, USA), with a tool nose radius of 0.4 mm, were used. The following tool geometry was applied: lead angle of 93°, both inclination angle and orthogonal rake angle equal to 0°. 

Another set of surfaces was prepared by mass finishing a part with an edge that was milled and honed. First, two sides were side-milled to create an edge at a corner, with an angle of approximately 90°. The two faces were then honed by hand, using emery paper to remove a burr left from milling. The first measurement of the edge was taken after the honing. The part was subsequently placed in a BelAir FMSL 8T series centrifugal disk mass-finisher (Bel Air Finishing Supply Corporation, North Kingstown, RI, USA) and then measured after finishing for 2.5 and again after 7.5 min. The abrasive media was R-1000, a polyester pyramid with a height of 6.4 mm, with zirconia particles embedded.

### 2.2. Analysis for Estimation of the 3D Curvature Based on Vectors Normal to Surface Patches

This analysis calculates curvature tensors at each scale in the data set and each location on the surface where there are a sufficient number of measured heights for the calculation. The calculated curvatures are considered to be constant over the triangular patches, which are the regions used for the curvature calculations. The range of scales available in a measurement goes from the sampling interval to the size of the measured region. 

**Figure 1:**
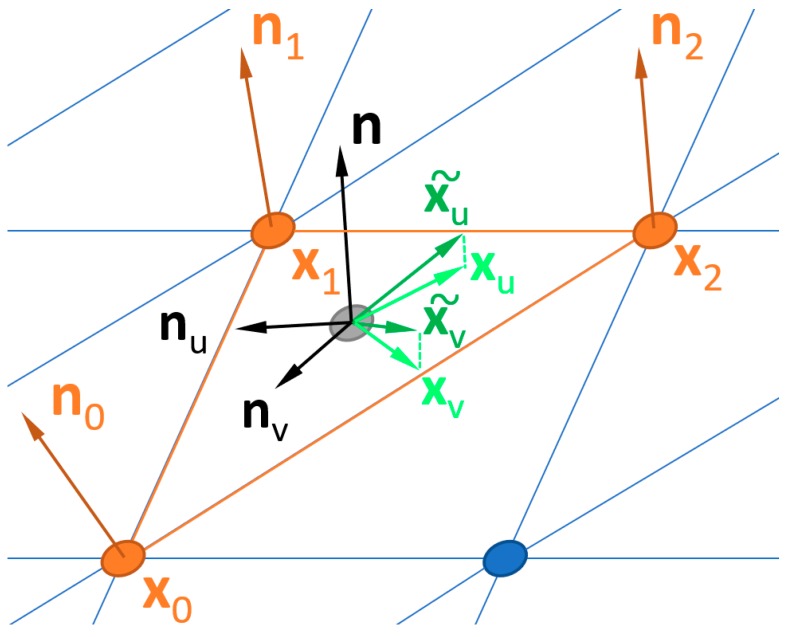
Visualization of **x***_u_*, **x***_v_*, **n***_u_* and **n***_v_* on a triangular patch.

First, three unit normal vectors are calculated, one for each vertex on the triangular patch that is used for the curvature tensor calculation (Figure 1). A covariance matrix method for computing unit normal vectors is used. At each vertex, the closest 3 × 3 neighborhood of measured heights is used for computing the normal vector. The edges of the measured region are excluded, due to insufficient measured heights, 2 × 2 or 3 × 2 neighborhoods of heights, instead of 3 × 3.

Next, using the Weingarten curvature tensor, **T**, a symmetric 3 × 3 matrix is calculated, assuming that the surface is continuous and everywhere differentiable (within the patch) [26]. The resulting eigenvalues are, *κ*_1_, *κ*_2_, 0, where the first two represent the principal curvatures. The resulting eigenvectors include **k_1_**, **k_2_**, the corresponding principal directions for the principal curvatures, and **n**, the unit normal vector for the triangular patch. 

Note that new local and global coordinate systems are introduced here for these calculations. Whereas measured, global heights were *z*(*x*,*y*), as is usual in the literature, these are represented below as *x*(*u*,*v*). Normal vectors are calculated from these global heights. Curvatures are calculated in a local coordinate system. This local coordinate system (*u*,*v*) is in the plane defined by the three points that constitute a single triangular patch. To calculate the curvature directions, transfers from the local systems (*u*,*v*) to the global (*x*,*y*) system are necessary.

At each scale, the surfaces that are considered here are completely defined by their partial directional derivatives and the partial directional derivatives of the unit normal vectors. Given the surface *x*(*u*,*v*) and its partials **x***_u_* and **x***_v_*, a unit normal vector **n** and its partials can be computed by the following formula:

(1) $$n=\frac{x_{u\;}\times \;x_{v}}{∥x_{u}\;\times \;x_{v}∥},\;n_{u}=\frac{\partial n}{\partial u},\;n_{v}=\frac{\partial n}{\partial v}$$

These four vectors have the following dependencies:

1. **x***_u_*, **x***_v_*, **n***_u_*, **n***_v_* are coplanar.
2. **n***_u_***x***_v_* = **n***_v_***x***_u_.*

The computation of **T** from **x***_u_*, **x***_v_*, **n***_u_*, **n***_v_* is a straightforward application of classical concepts of differential geometry [26]. The coefficients of the first and second fundamental form, or shape, tensor can be calculated as:

(2) $$E=x_{u}\cdot x_{u},\;F=x_{u}\cdot x_{v},\;G=x_{v}\cdot x_{v},$$

(3) $$L=-n_{u}\cdot x_{u},\;M_{1}=-n_{u}\cdot x_{v},$$

(4) $$M_{2}=-n_{v}\cdot x_{u},\;N=-n_{v}\cdot x_{v}.$$

Then the Weingarten curvature matrix can be created,

(5) $$W=\left(\begin{array}{cc} \frac{LG-M_{1}F}{EG-F^{2}} & \frac{LG-M_{1}F}{EG-F^{2}}\\ \frac{LG-M_{1}F}{EG-F^{2}} & \frac{LG-M_{1}F}{EG-F^{2}} \end{array}\right)$$

with its eigenvalues *κ_*1*_*, *κ_*2*_* and its corresponding eigenvectors:

(6) $$w_{1}=\left(\begin{array}{c} w_{11}\\ w_{12} \end{array}\right),\;w_{2}=\left(\begin{array}{c} w_{21}\\ w_{22} \end{array}\right).$$

The eigenvalues are used to calculate the Gaussian curvature, *K*, and the mean curvature, *H*; and the eigenvectors are used to calculate the principal directions **k**_1_ and **k**_2_ as it follows:

(7) $$K=\kappa_{1}\kappa_{2},\;H=\frac{1}{2}(\kappa_{1}+\kappa_{2}),$$

(8) $$k_{1}=w_{11}x_{u}+w_{12}x_{v},\;k_{2}=w_{21}x_{u}+w_{22}x_{v}.$$

Having all necessary components, curvature matrix **T** can be constructed:

(9) $$T=PDP^{-1},$$

where $P=(k_{1},k_{2},n)$ and,

(10) $$D=\left(\begin{array}{ccc} \kappa_{1} & 0 & 0\\ 0 & \kappa_{2} & 0\\ 0 & 0 & 0 \end{array}\right).$$

Theisel et al. [16] presented a new technique for estimating curvature tensor **T** in a triangular mesh. That method shows better error behavior than a cubic fitting [13] and is independent of rotations of the mesh and does not involve any parameterization or fitting. The accuracy of Theisel’s method depends primarily on the accuracy of the estimation of the unit normal vectors, which was the first part of the curvature computations above. 

In that normal approach, only a single (non-degenerate) triangle, with the vertices *x_*0*_*, *x_*1*_*, *x_*2*_* and the corresponding normals *n_*0*_*, *n_*1*_*, *n_*2*_*, are considered (see Figure 1). A point and normal vector on the triangle can be obtained by applying linear interpolation in local coordinates (*u*,*v*), with the origin *x_*0*_* and the base vectors *x_1_ − x_*0*_* and *x_*2*_* – *x_*0*_*:

(11) $$\tilde{x}=\tilde{x}(u,v)=x_{0}+u(x_{1}-x_{0})+v(x_{2}-x_{0}),$$

(12) $$\tilde{n}=\tilde{n}(u,v)=n_{0}+u(n_{1}-n_{0})+v(n_{2}-n_{0}),$$

The idea that stands behind the introduction of interpolated $\tilde{x}$ and $\tilde{n}$ is to use them for calculating vectors **x***_u_*, **x***_v_*, **n***_u_*, **n***_v_* and, subsequently, curvature matrix **T**. Unit normal vectors and their derivatives can be computed following Theisel et al. [16]:

(13) $$n\left(u,v\right)=\frac{\tilde{n}}{∥\tilde{n}∥},\;n_{u}=\frac{\partial n}{\partial u},\;n_{v}=\frac{\partial n}{\partial v}$$

For the partials of the surface, we can obtain:

(14) $${\tilde{x}}_{u}\left(u,v\right)=\frac{\partial \tilde{x}}{\partial u}=x_{1}-x_{0},\;{\tilde{x}}_{v}\left(u,v\right)=\frac{\partial \tilde{x}}{\partial v}=x_{2}-x_{0}$$

In order to assure that condition 1 (**x***_u_*, **x***_v_*, **n***_u_*, **n***_v_* are coplanar) is met, ${\tilde{x}}_{u}$ and ${\tilde{x}}_{v}$ are projected onto the plane defined by **n***_u_* and **n***_v_*:

(15) $$x_{u}={\tilde{x}}_{u}-(n{\tilde{x}}_{u})n,\;x_{v}={\tilde{x}}_{v}-(n{\tilde{x}}_{v})n.$$

Now, the curvature matrix **T** can be computed, by applying Equations (13)–(15) into Equations (2)–(10).

### 2.3. Multiscale Curvature Characterization Analysis

Multiscale characterizations can be achieved in several ways [2]. Two different methods of specifying the scale of the curvature analyses are described here. These two methods both apply to the selection of the measured heights that are used for the estimation of the normal vectors and to the selection of three points that form the triangular patches. 

**Figure 2:**
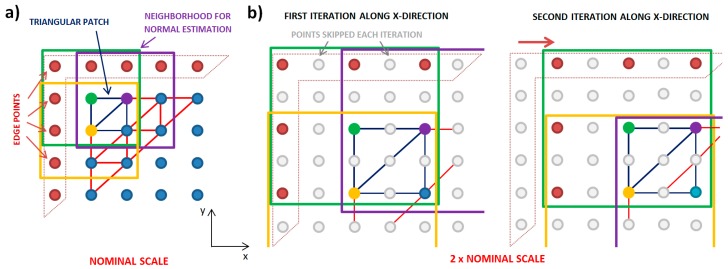
Down-sampling, one method of the multiscale analysis: (**a**) at the nominal scale, which is equal to the sampling interval, (**b**) at a two times the nominal scale. The colors of the squares, green, yellow, and purple, correspond to the color of the points that center the vertices of the triangles. Edge points are colored with red.

The first multiscale method here is down-sampling, shown in Figure 2. In this down-sampling, more measured heights are skipped with each iteration of the multiscale calculations, in order to achieve increasingly larger scales. At the finest, or nominal, scale, the spacing is the sampling interval. At two times the nominal scale, the spacing it is twice the sampling interval, for which every other measured height is used. At three times the sampling interval, every third measured height would be used. The scale here is the length of the horizontal interval in *x* and *y*, between the measured heights used in each analysis for determining the curvature. 

The down-sampling is applied for both the selection of the measured heights for calculating the normal vectors and for selecting the points that define the triangular patches for the eigenvalue problem. To determine the position for calculating the curvature, even at the large scales, the iterations are performed at each location. That is, the calculation is indexed horizontally, one sampling interval for each locational calculation.

After heights are skipped in this down-sampling routine, just the nine, not-skipped, measured heights are used to calculate the normal unit vectors at each scale. These are the eight heights closest to the apexes of the triangular patches (Figure 2a) and the central point (apex) used for the eigenvalue problem. The spacing between the heights is scale-dependent and increases with the scale (Figure 2b). These triangles are always equilateral, right triangles in projection on a horizontal plane. The projected length of the short sides are equal to the scale. More details of the method and its application for multiscale analysis can be found in work by Bartkowiak and Brown [27]. The calculation of the curvature tensor is done for the next location distant from the previous, by using the original sampling interval (Figure 2b). 

**Figure 3:**
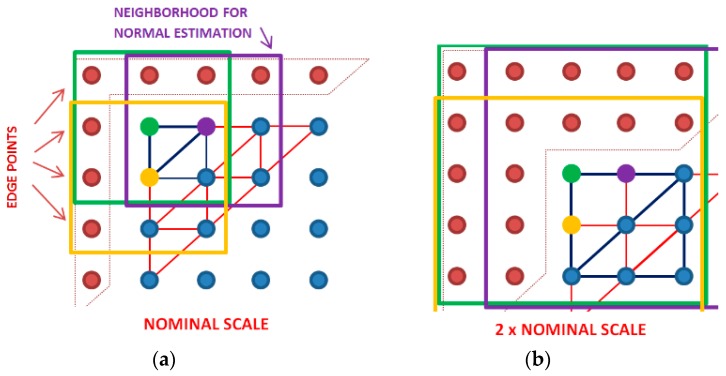
The second method of the multiscale analysis: (**a**) at the nominal scale, which is equal to the sampling interval, (**b**) at a two times the nominal scale.

In the second, multiscale method considered here, no in-between heights are skipped in normal estimation, while the values for the scales are determined identically to the first method (Figure 3). In this way, each iteration by both methods includes the same measured regions, and they are signified by the same scale. The second method uses all the measured heights in the neighborhood, instead of just nine. The size of a neighborhood changes with scale. For the nominal scale, both methods use the same measured heights for the calculation of the unit normal vectors. For larger scales, the number of heights grows with the multiplication of the original sampling interval *s*, so that the neighborhood consists of (1 + 2*s*) × (1 + 2*s*) points. For instance, for a scale equal to three times the sampling interval, it is necessary to consider 7 × 7 heights, for calculating the unit normal vector for an apex that is centrally placed inside the neighborhood.

In both methods, the normal unit vectors, and curvatures, are not estimated along the edges of the measured region, where entire neighborhoods cannot be formed. This second method can be time-consuming for the larger scales, because the covariance calculation includes more points.

## 3. Results

In the following sections, renderings of the measurements of the studied surfaces are shown at different scales. The calculated curvatures and Gaussian and mean curvatures are shown as a function of position and are plotted versus scale, along with the standard deviations. 

### 3.1. Prepared Edge, Honed and Mass-Finished

**Figure 4:**
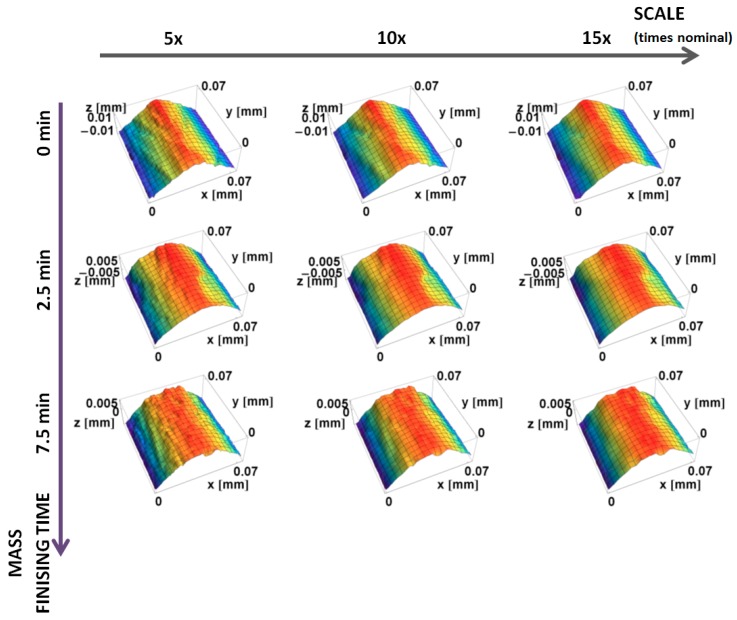
Renderings of topographic measurements of the edge machined and honed (0 min), and after 2.5 and 7.5 min of mass finishing, at three scales: 5× original sampling interval (1.25 µm), 10× (2.50 µm), and 15× (3.75 µm).

Renderings of topographic measurements with three downsized scales of the machined and honed edge, as well as two finishing times, are presented in Figure 4. With the increasing scale, surfaces appear smoother, because the fine-scale details are skipped in the down-sampling. 

**Figure 5:**
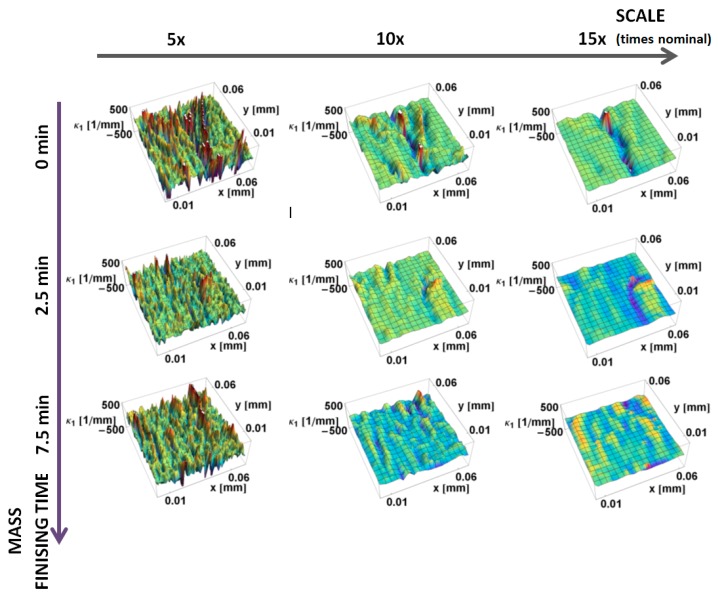
Maximum principal curvature, *κ*_1_, on the prepared edge as a function of position, calculated using the down-sampling method for three scales.

The principal, *κ_*1*_*, curvatures, those with the largest magnitude, calculated by the down-sampling method, are shown as a function of position on the surface and the scale of calculation in Figure 5. Convex curvatures are negative and concave are positive, as usual. 

At the largest scales, there is little variation in curvature. The magnitudes of the curvatures tend to increase with decreasing scale. Many small regions of convexity at the smallest scales are evident in Figure 5. The curvature of the prepared edges cannot be discriminated at the finest scales. At these fine scales, a multitude of fine features, with large principal *κ_*1*_* curvatures, masks the curvature of the edge. The curvatures on the honed part are clearly visible at 10× nominal scale. The curvature of the part that was mass-finished for 2.5 min is relatively uniform at the largest scales (10× and 15×). Concave features are clearly visible at 15×. The effect of mass-finishing is evident at all three scales. 

**Figure 6:**
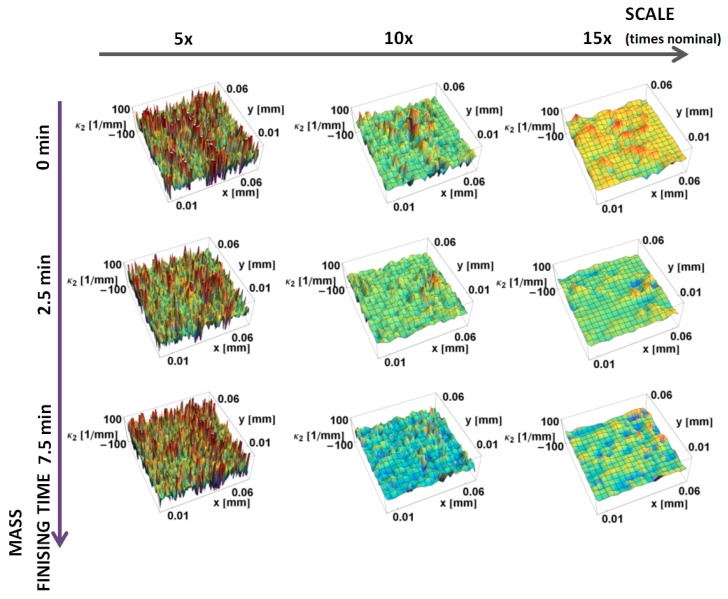
Minimum principal *κ*_2_ curvature, as on the prepared edges, a function of position calculated, using the down-sampling method for three scales.

The principal *κ_*2*_* curvatures, minimum in magnitude, calculated by the down-sampling method, are shown as a function of position on the surface and the scale of calculation in Figure 6. Similar to *κ_*1*_* at the large scales, there is little variation in curvature. The magnitudes of the curvatures tend to increase with decreasing scale. A positive curvature region is evident around the manufactured edge at 5× and 10× nominal scale. However, its value is significantly lower than the *κ_*1*_* curvature. The mass finishing process decreases the magnitude of *κ_*2*_* curvature for all three scales shown.

**Figure 7:**
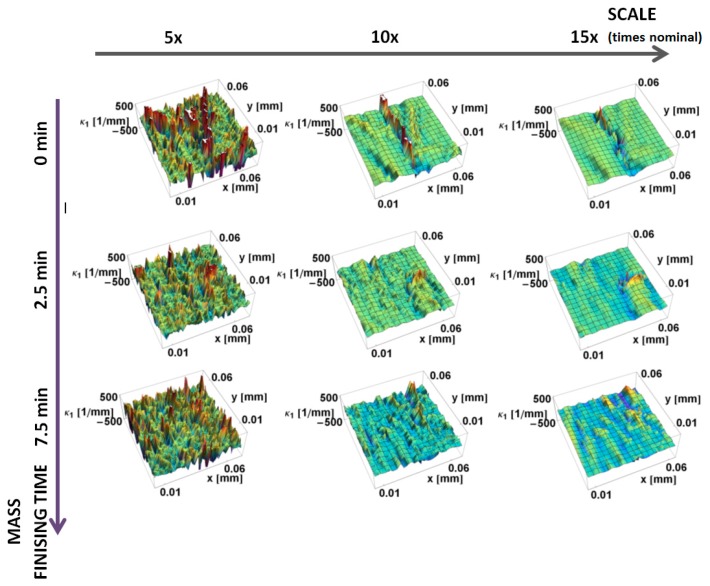
Maximum principal curvature, *κ*_1_, on the prepared edge, as a function of position calculated, using the increasing neighborhood method, for three scales.

**Figure 8:**
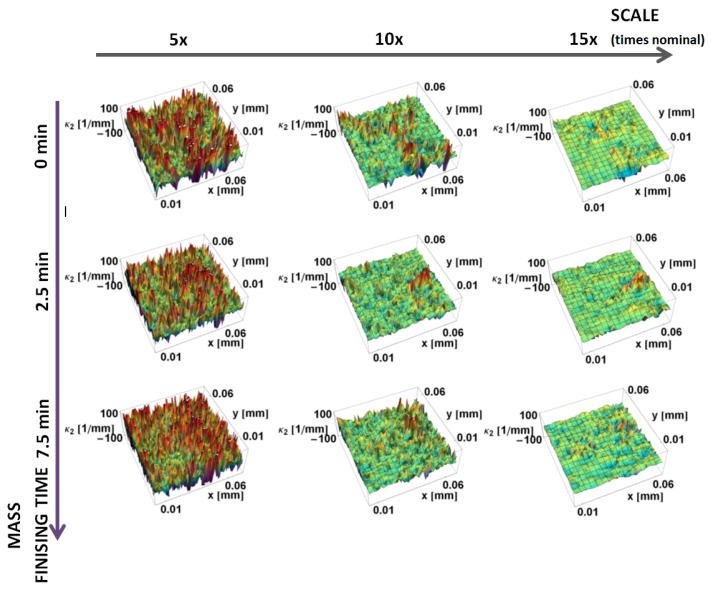
Minimum principal curvature, *κ*_2_, on the prepared edge as a function of position calculated, using the increasing neighborhood method for three various scales.

The principal *κ_*1*_* and *κ_*2*_* curvatures, calculated by the increasing neighborhood method, are shown as a function of position on the surface and the scale of calculation in Figure 7 and Figure 8, respectively. These show the same trends as the down-sampling method. The variability of curvatures for finer scales is higher for the increasing neighborhood method, when comparing the same scales. For the same subregions, both minimal and maximal curvature take greater values. For larger scales, more points are used in the calculation of the normal vectors, so artifacts and fine-scale features influence more points in their normal vectors estimation, which make these results more sensitive to local variations. The expected smoothing effect is less evident when compared to the down-sampling method.

**Figure 9:**
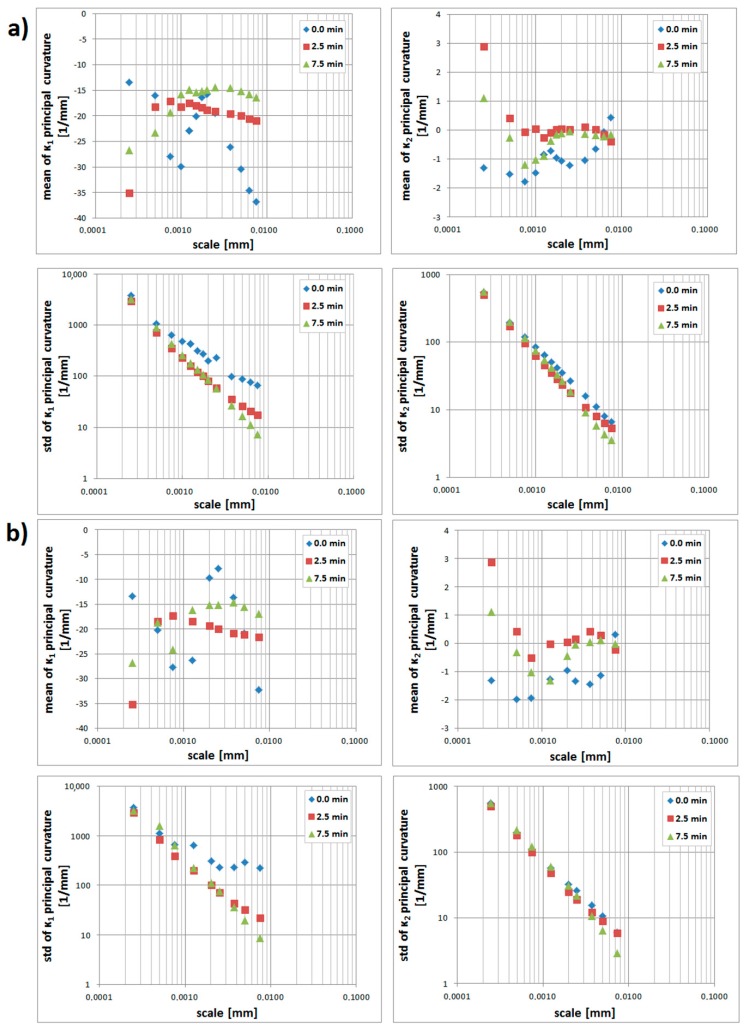
Statistics on the prepared edge as a function of scale: (**a**) mean and standard deviation of *κ*_1_ and *κ*_2_ principal curvatures, calculated for three scales by down-sampling, (**b**) mean and standard deviation (std, in short) of *κ*_1_ and *κ*_2_ principal curvatures, calculated for three scales by increasing the neighborhood.

Mean and standard deviations of the principal curvatures on the prepared edges, calculated by the down-sampling method, are shown, versus the log of the scale, in Figure 9a. The mean values of principal curvatures change with the scale for all the surfaces. The standard deviation of principal curvatures decreases regularly with increasing scale, i.e., the distribution of the curvatures is distinctly varied at the finer scales. For larger scales, the dispersion measure decreases, as fewer fine-scale surface features, characterized by high curvature, become evident, and mean values of principal curvature tend to indicate the general shape of the edge. The curvatures of microfeatures are generally greater than the overall form or waviness, which is quantified as larger values of standard deviations in comparison with the mean. It appears that the mean *κ_*1*_* discriminates the edges for scales greater than 4µm, with a sufficient sample size, because the variance is large. Mean values of *κ_*1*_* are negative for all calculated scales, which is consistent with the overall convexity of the surface perpendicular to the prepared edge. Logically, the means of the minimum principal curvatures, *κ_2,_* are smaller. Their proximity to zero, particularly at large scales, is consistent with the straightness of the surfaces parallel to the prepared edge.

Statistics calculated by the method are presented in Figure 9b. It appears that the mean and standard deviations of *κ_*1*_* might be used to discriminate the prepared edges for some scales between 4 and 10 µm. The greatest differences between those two methods appears at the greatest scales. Similar trends appear for *κ_*2*_*. Standard deviations of maximal curvatures take greater values when calculated by increasing the neighborhood method, which supports the effect of microfeatures that is evident with growing scales.

### 3.2. Cutting Tool Edge and Turned Surfaces

**Figure 10:**
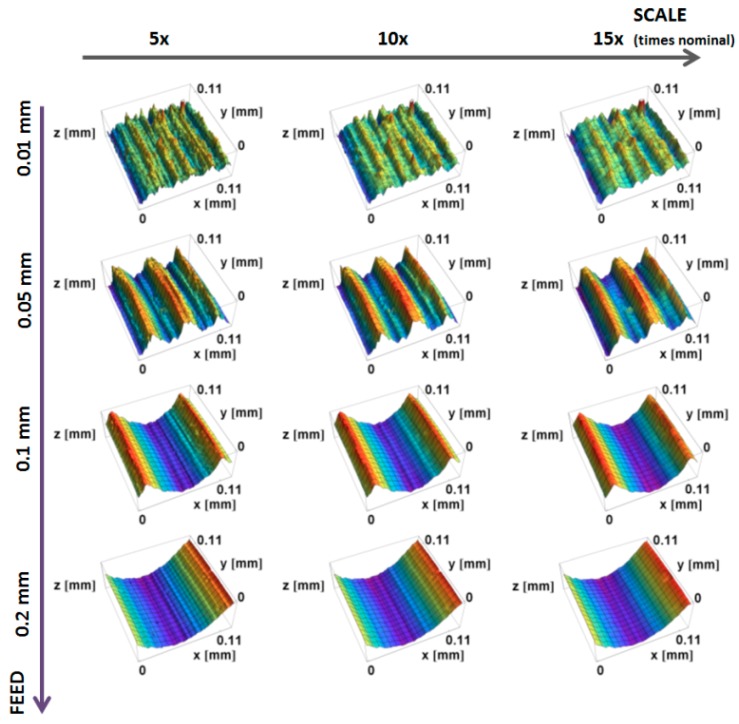
Renderings of surfaces turned at four feed rates, at three scales.

Renderings of measurements representing three downsized scales and feed rates of the prepared surfaces and tool edge are presented in Figure 10. The figure presents surfaces for various scales. Both the turned surfaces and the tool edges are examples of clearly anisotropic textures. With increasing scales, fine-scale ridges and valleys tend to be smoothed. Both principal curvatures were calculated for the prepared region of 700 × 700 heights. 

**Figure 11:**
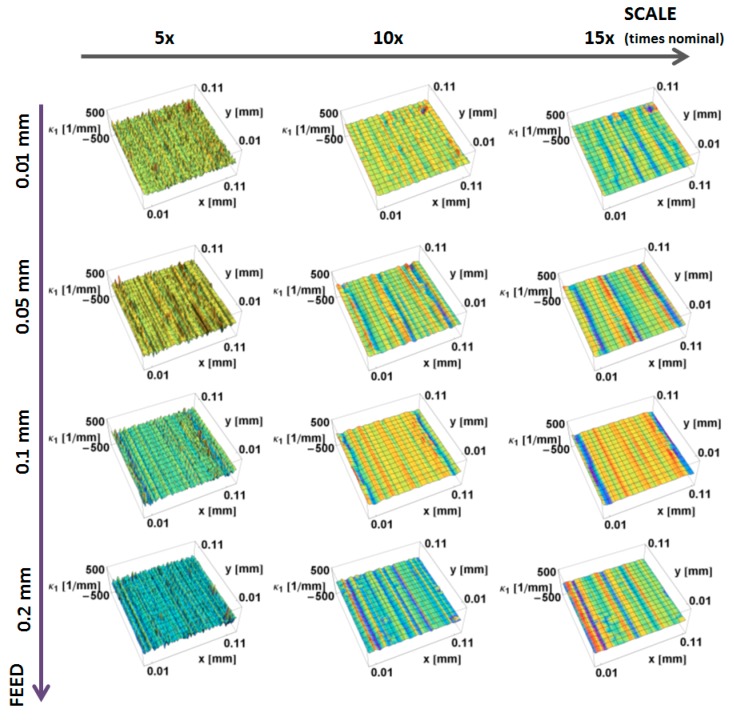
Maximum principal curvature, *κ*_1_, as a function of position calculated for turned surfaces, using the down-sampling method for three scales.

The principal, *κ_*1*_*, curvatures, those with the largest magnitudes, calculated by the down-sampling method, are shown as a function of position on the surface and the scale of calculation in Figure 11. Similar to the mass finished surfaces, at the largest scales the variation in curvature decreases. The magnitudes of the curvatures have a tendency to increase with decreasing scale. The three top ridges on the surface, machined at 0.05 mm/rev, and the two ridges for the surface, machined at 0.1 mm/rev, are evident as blue stripes at larger scales. At finer scales, the anisotropic character of all of the machined surfaces becomes less visible. At finer scales, a multitude of fine features, with large principal *κ_*1*_* curvatures, masks the directional features. The curvatures of the main valleys are more evident at larger scales for surfaces machined at 0.05, 0.1 and 0.2 mm. 

**Figure 12:**
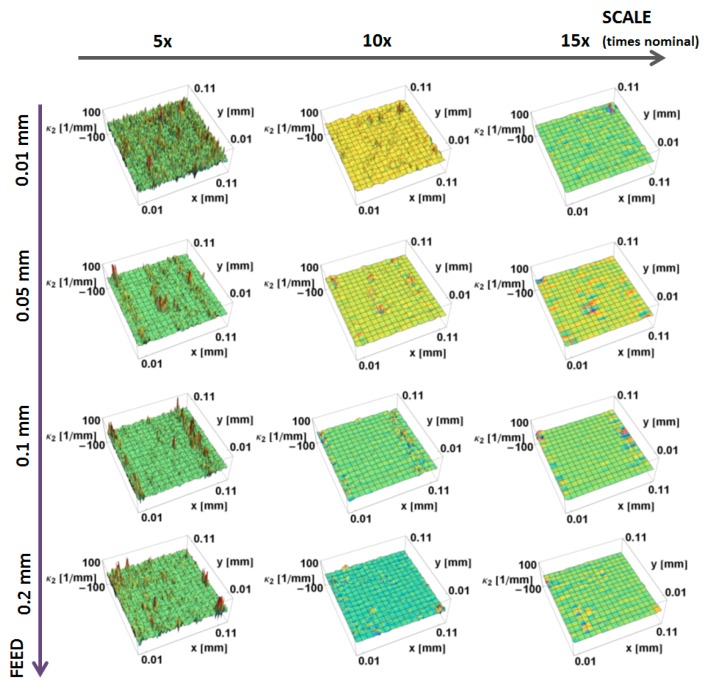
Minimum magnitude of the principal curvatures, *κ*_2_, as a function of position for turned surfaces calculated, using the down-sampling method, for three scales.

The principal *κ_*2*_* curvatures, minimum in magnitude, calculated by the down-sampling method, are shown as a function of position on the surface and of the scale of calculation in Figure 12. Similar to *κ_*1*_* at the large scales, there is little variation in curvature. The magnitudes of the curvatures tend to increase with decreasing scale. At finer scales, the directional nature becomes more visible at the ridges, appearing as lines of high-magnitude curvature. At larger scales, for all the measured surfaces, the minimum curvature tends to zero, indicating that the surface is flat in one direction. The feed rate influences the magnitude of the *κ_*2*_* curvature at all three of the scales shown. 

**Figure 13:**
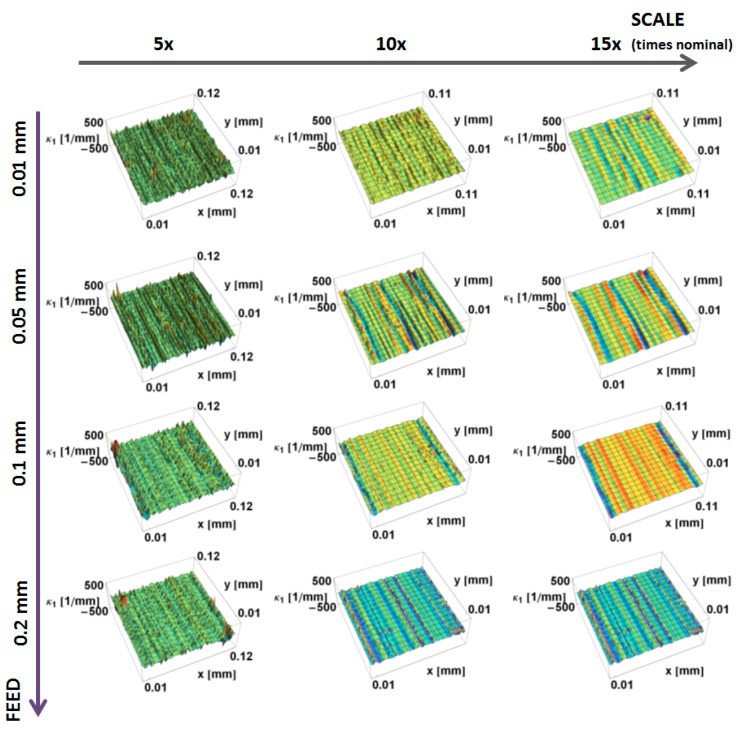
Maximum principal curvature, *κ*_1_, as a function of position for turned surfaces, using the increasing neighborhood method for three scales.

**Figure 14:**
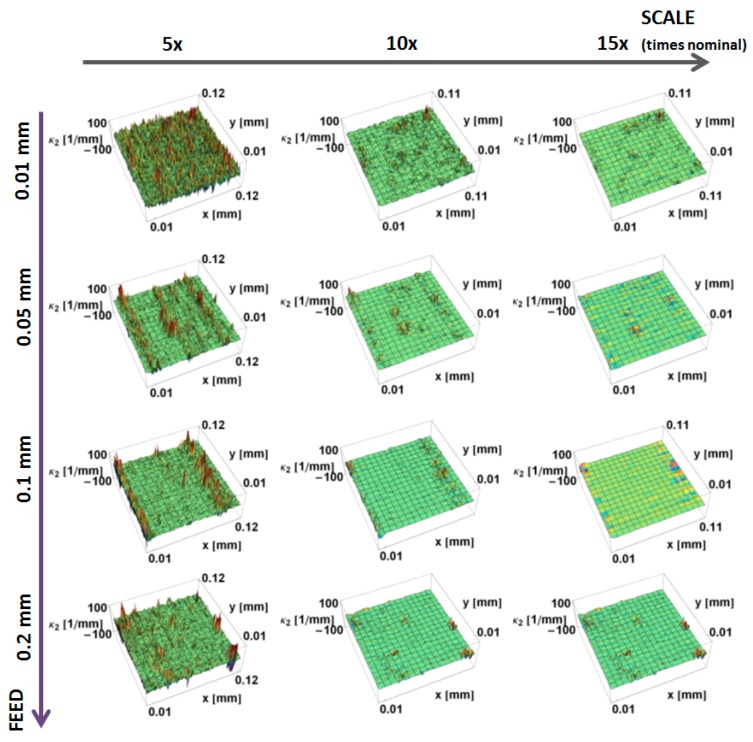
Minimum principal curvature, *κ*_2_, as a function of position for turned surfaces, using the increasing neighborhood method for various scales.

The principal *κ_*1*_* and *κ_*2*_* curvatures, calculated by the increasing neighborhood method, are shown as a function of position on the surface and the scale of calculation in Figure 13 and Figure 14, respectively. These show the same trends as the down-sampling method. As in the previously studied surfaces, at larger scales, more heights are used in the calculation of the normal vectors, which makes these results more sensitive to artifacts and local variations. For the same subregions, principal curvatures take greater values for the increasing neighborhood method, and the smoothing effect is less evident. 

**Figure 15:**
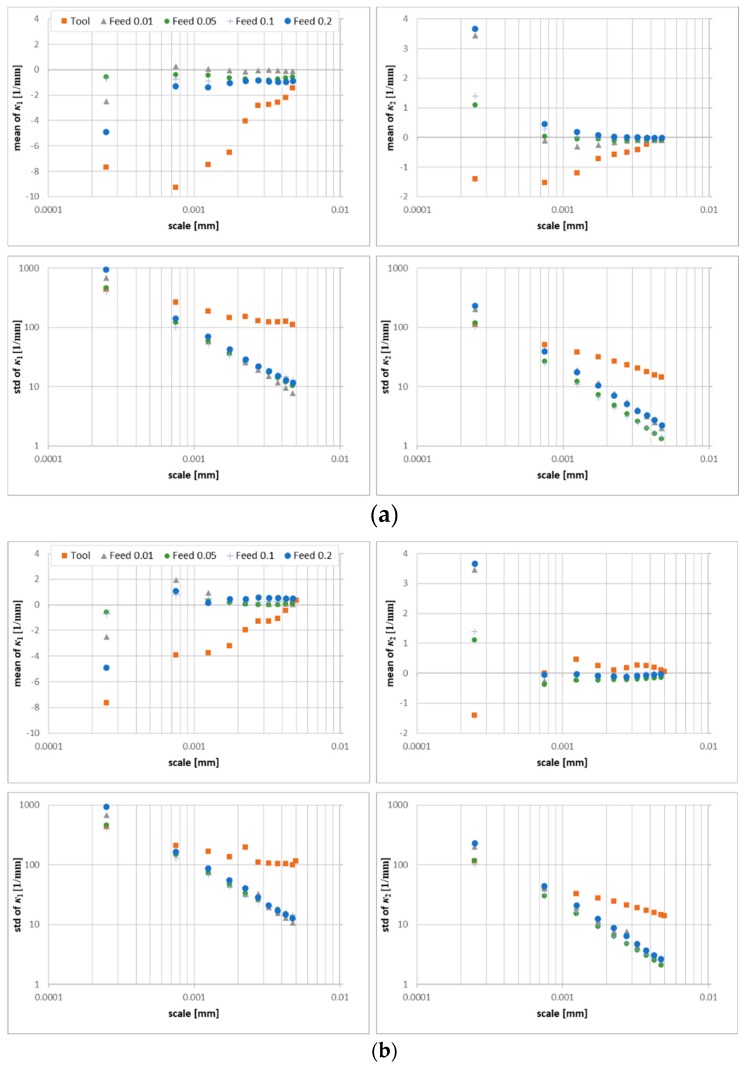
Statistics on the turned surfaces and tool as a function of scale: (**a**) mean and standard deviation of *κ*_1_ and *κ*_2_ principal curvatures, calculated for three scales by down-sampling, (**b**) mean and standard deviation of *κ*_1_ and *κ*_2_ principal curvatures calculated for three scales by increasing the neighborhood.

Mean and standard deviations of the principal curvatures on the prepared edges, calculated by the down-sampling method, are shown versus the log of the scale in Figure 15a. The mean values of the principal curvatures change with the scale for all the surfaces. The standard deviations of the principal curvatures decrease regularly with increasing scale, i.e., the variance of the curvatures is larger at the finer scales. The mass-finished edge shows the same tendency.

The maximum curvatures decrease linearly (R^2^ > 0.93) with feed rate, for scales between 0.75 and 1.25 µm. Other scales show the same trend, although the correlations are weaker (0.42 > R^2^ > 0.67). In addition, the mean *κ_*1*_* discriminates the surfaces for scales between 0.75–1.25 µm, where the variance is large. 

The mean and standard deviations of the minimum principal curvatures, *κ_*2*_*, are smaller than for maximum curvature, *κ_*1*_*. Their proximity to zero, particularly at large scales, is consistent with the straightness of the surfaces that are parallel to the prepared edge.

Standard deviations of both principal curvatures show mediocre to poor correlation with feed rate (R^2^ < 0.72 for maximum and R^2^ < 0.17 for minimum), suggesting that the variance of curvatures is not influenced strongly by feed. The strongest correlations were observed between the feed rates and the mean minimum curvatures (R^2^ > 0.8 for scales between 0.75 and 3.25 µm, with a maximum of 0.982 at 2.75 µm). This suggests that minimum curvature is feed-dependent. 

Statistics calculated by the method are presented in Figure 15b. It appears that the mean and standard deviations of *κ_*1*_* might be used to discriminate the prepared edges for some scales between 2.25 and 4.75 µm. The coefficients of determination R^2^ for regression analysis for the same range take values greater than 0.81. These means of minimum curvatures correlate more weakly than when they are calculated using the down-sampling method (maximum R^2^ = 0.74). The greatest differences in the statistical parameters between those two calculation methods appears at the largest scales. Similar trends appear for *κ_*2*_*.

For both methods, the mean of principal curvatures of the tool edge take similar values for larger scales. The variation of the curvatures for the tool edges is greater than in the resulting turned surfaces. This suggests that large-scale features on the tool edge are transferred to the machined surface, whereas fine-scale details on the tool are not.

## 4. Discussion

The viability of this method of calculation of unit normal vectors and applying eigenvalue analyses to areal topographic measurements has been clearly demonstrated for all the variations studied. The results are consistent with the expected curvatures and tendencies with scale, feed, and mass-finishing times on the manufactured surfaces studied here. Curvatures in 3D can be calculated as a function of position and scale directly from a regular spatial array of heights. The presented method requires estimation of unit vectors normal to the surface prior to estimation of the curvature tensor. The method is sensitive to the quality of the estimation of the normal vectors. Two calculation methods present similar results for multiple scales; however, they vary in the computational time. The smoothing effect is less evident for the increasing neighborhood method, which can suggest its potential for discrimination. The down-sampling method takes, on average, 1/10 of the time in comparison with the increased-neighborhood method. 

At the finer scales, the principle curvatures tend to increase with decreasing scale for all measured surfaces, because fine-scale details have larger magnitude curvatures. The principal curvatures calculated for particular scales tend to decrease with time in the mass finisher. The feed rate influences the curvature of the resulting topographies. In the cases studied, the mean of values for the principal curvatures are more appropriate for discrimination and correlation than standard deviations.

The important criterion for adding value with texture characterization methods is their ability to correlate with some phenomena of interest, such as processing or performance. The inclusion of multiscale methods in a characterization and analysis provides an important dimension for improvement, as has been demonstrated many times previously [2,9,28,29]. Some surface topographies cannot be well represented by smooth functions at all, or any, scales. Thus, a method of curvature calculation that does not require fitting smooth geometries, like quadratic forms, might be appropriate. The same can be said for the profiles. 

The lengths of irregular profiles change with scale, as do the slopes [30]. Not surprisingly, then, the curvature also changes with scale and, naturally, with position, also. As with the area-scale analysis, correlations between curvature and topographically related behavior or processing might only be found over a narrow range of scales. Determination of appropriate scales for strong correlations and confident discriminations can follow the method previously reported for scale-based correlation, using multiscale regression and discrimination tests [9]. Multiscale characterizations of curvature have the potential to enable new, strong correlations and confident discriminations. 

A recent study involving ENS-Cachan (Ecole normale supérieure de Cachan) and WPI (Worcester Polytechnic Institute) showed that multiscale analysis of curvatures of profiles could be successful for determining fatigue limits [9]. This is logical, because positive curvatures relate to stress concentrations that increase the likelihood of crack initiation. In addition, curvature has appeal as an appropriate geometric characterization for many kinds of contact mechanics. Because the curvature changes with position, appropriate statistical characterizations must be used as well [2].

Whitehouse [31] discusses sensitivity to scale and notes that it should be four to five times greater than the sampling interval. He also proposes that a better approximation can be found using a seven-point average to determine the slopes for the first step of the double-difference method. However, the sampling interval is often dictated by the measurement instrument, and it can be somewhat arbitrary with regard to the scale of the topographically related interactions of interest. A better approach could be to examine all the scales available in the measurement. Subsequently, these can be compared for regression and discrimination tests as a function of scale. This can lead to identification of the scales of interaction for the phenomena of interest.

The richness of the multiscale tensor curvature characterizations suggest that they have a strong potential for many kinds of applications in engineering, forensics, paleontology, physical anthropology, and archaeology.

## 5. Conclusions

The viability of these methods has been demonstrated for multiscale characterization of curvature tensors on measured topographies (*z = z(x*,*y)*). The analyses are based on calculating unit-normal vectors to the surface, at three proximal locations, and then using an eigenvalue approach to the problem of calculating the curvature tensors. The curvature tensors of measured topographies can be calculated and studied over a range of scales and positions. These methods are useful and feasible, and they have been demonstrated successfully for a variety of surfaces. 

The validity of these methods has been furthered by the demonstrable consistency of the results with expectations on manufactured surfaces. These expectations include the nature of the curvature as shown by principal curvature values and their orientations relative to manufactured features on the surfaces. Mean curvature values and variance provide further validation of these methods by meeting expectations. The multiscale analyses and resulting multiscale characterization, using curvature tensors on areal measurements of topographies, also meets with the expectations based on the manufactured features. 

The two methods studied here, for calculating the unit-normal vectors on the surface and systematically adjusting the scale of calculation, show the expected differences. The increasing neighborhood method, i.e., the second method, in contrast to the down-sampling method, has been shown to lead to a decrease in the variation of the curvatures. This suggests that the neighborhood method could be valuable when there is a concern that irregularity in the topographic data might be masking interesting tendencies. This comparison, which is consistent with expectations, demonstrates the viability and furthers the demonstration of the validity of both of these methods.

## 6. Patents 

The multiscale curvature analysis in terms of outlier removal is the subject of patent application: Measurement equipment with outlier filter, US20180038687A1.
